# Predicting post-radiation genitourinary hospital admissions in patients with localised prostate cancer

**DOI:** 10.1007/s00345-022-04212-y

**Published:** 2022-11-10

**Authors:** Rowan David, Mrunal Hiwase, Arman A. Kahokehr, Jason Lee, David I. Watson, John Leung, Michael E. O‘Callaghan

**Affiliations:** 1 College of Medicine and Public Health, Flinders University, Bedford Park, South Australia Australia; 2 Department of Urology, Flinders Medical Centre, SA Health, Bedford Park, 5042 Australia; 3 GenesisCare, Adelaide, Australia; 4 South Australian Prostate Cancer Clinical Outcomes Collaborative, Adelaide, Australia; 5 Discipline of Medicine, Freemasons Foundation Centre for Men‘s Health, University of Adelaide, Adelaide, Australia

**Keywords:** Prostate cancer, Radiotherapy, Radiation therapy, External beam radiotherapy, Genitourinary complications, Hospital admission, Genitourinary toxicity, Hospitalisation, Machine learning, Decision curve analysis

## Abstract

**Purpose:**

The risk of treatment-related toxicity is important for patients with localised prostate cancer to consider when deciding between treatment options. We developed a model to predict hospitalisation for radiation-induced genitourinary toxicity based on patient characteristics.

**Methods:**

The prospective South Australian Prostate Cancer Clinical Outcomes registry was used to identify men with localised prostate cancer who underwent curative intent external beam radiotherapy (EBRT) between 1998 and 2019. Multivariable Cox proportional regression was performed. Model discrimination, calibration, internal validation and utility were assessed using C-statistics and area under ROC, calibration plots, bootstrapping, and decision curve analysis, respectively.

**Results:**

There were 3,243 patients treated with EBRT included, of which 644 (20%) patients had a treated-related admission. In multivariable analysis, diabetes (HR 1.35, 95% CI 1.13–1.60, *p* < 0.001), smoking (HR 1.78, 95% CI 1.40–2.12, *p* < 0.001), and bladder outlet obstruction (BOO) without transurethral resection of prostate (TURP) (HR 7.49, 95% CI 6.18–9.08 *p* < 0.001) followed by BOO with TURP (HR 4.96, 95% CI 4.10–5.99 *p* < 0.001) were strong independent predictors of hospitalisation (censor-adjusted c-statistic = 0.80). The model was well-calibrated (AUC = 0.76). The global proportional hazards were met. In internal validation through bootstrapping, the model was reasonably discriminate at five (AUC 0.75) years after radiotherapy.

**Conclusions:**

This is the first study to develop a predictive model for genitourinary toxicity requiring hospitalisation amongst men with prostate cancer treated with EBRT. Patients with localised prostate cancer and concurrent BOO may benefit from TURP before EBRT.

**Supplementary Information:**

The online version contains supplementary material available at 10.1007/s00345-022-04212-y.

## Introduction

Prostate cancer is the second most common malignancy amongst men worldwide, and the number of long-term prostate cancer survivors continues to increase. [1] Prostate cancer is often treated with radiotherapy or surgery, with similar local control outcomes but different treatment-related toxicity profiles and side effect profiles. [2] However, there are limited high-quality data identifying predictive factors for genitourinary toxicity after radiotherapy.

The development of genitourinary toxicity following EBRT has been demonstrated to be influenced by a range of factors other than dosimetric variables alone [3, 4] and include baseline urinary symptoms [5, 6] and comorbidities such as diabetes [7]. However, predictive models classically have been limited to mechanistic analysis of dose-volume metrics [8, 9], which are often already incorporated into radiotherapy delivery planning systems.

This study used pre-treatment clinical factors to develop and validate a novel predictive model for radiotherapy-related genitourinary toxicity requiring hospital admission and then determined the clinical utility of the model by using decision curve analysis.

## Material and methods

### Participants

The prospective South Australian Prostate Cancer Clinical Outcome Collaborative (SA-PCCOC) registry was used to identify men with localised prostate cancer who underwent local curative intent external beam radiotherapy between January 1, 1998, and January 31, 2019. The SA-PCCOC registry prospectively recruits > 90% of patients who are diagnosed with prostate cancer in the State of South Australia. We linked patient records from the SA-PCCOC registry with the Integrated South Australian Activity Collection (ISAAC) Hospital Administrative Database to identify patients who presented to any major hospital in South Australia with treatment-related genitourinary toxicity, as defined by a pre-selected list of International Classification Disease 10th Edition (ICD-10-AM)/ Australian Classification of Health Interventions (ACHI). Data linkage was performed by matching patient identifiers within ENVIDO, South Australia. The list of admission and procedures codes were selected based on the literature [10] and recommendations from a multidisciplinary panel, including a urologist, radiation oncologist, general surgeon and a clinical epidemiologist (Supplementary Table 1). A range of genitourinary toxicity events were analysed, including haematuria, irradiation cystitis, urethral stricture, urinary incontinence and urinary retention (Supplementary Table 2).

#### Primary outcomes

Of the identified patients with prostate cancer, baseline characteristics, including age (continuum), Charlson Comorbidity Index (continuum, 0/1–2/3–4/ > 4), diabetes mellitus (present/absent), hypertension (present/absent), smoking history (present/absent), bladder outlet obstruction (yes/ no), transurethral resection of the prostate (TURP) before radiotherapy (yes/ no), were extracted. Patients were further categorised as having bladder outlet obstruction (BOO) with or without TURP prior to EBRT. Genitourinary toxicity event-free survival (EFS) rates were then determined and compared between patient groups at increased risk of treatment-related GU toxicity.

#### Secondary outcomes

Treatment-related factors, including dose (grey; continuum and > 80 Gy vs ≤ 80 Gy), fractionation and date of treatment completion (< 2009 vs ≥ 2009), were extracted. Oncological characteristics, including T-stage (T1 vs T2 vs T3), Gleason score (< 7 vs 3 + 4 vs 4 + 3 vs > 7 and continuum) and baseline prostate-specific antigen (PSA; continuum) level, were also extracted. The admission data were separated into patients who received EBRT < 2009 and ≥ 2009 to account for the use of three-dimensional conformal radiation therapy (3DCRT) and intensity-modulated radiotherapy/volumetric-modulated arc therapy (IMRT/VMAT), respectively.

#### Statistical analysis

Relationships between genitourinary toxicity-related hospital admission and patient, tumour or treatment characteristics were analysed using multivariable Cox proportional hazard regression analysis. Regression analysis results are presented as a hazard with a 95% confidence interval. Missing clinical data were replaced using multiple imputations by chained equations before regression analysis (Supplementary Fig. 1).

The model development process was conducted following the TRIPOD checklist. [11] Multivariable model development used a backward elimination variable selection process with two-sided alpha = 0.05. [12] Collinearity among the variables was assessed using correlation coefficients. Diabetes was selected rather than the Charlson comorbidity score to reduce multicollinearity in the multivariable analysis. Model validation was performed by the ABCD approach put forward by Steyerberg et al. [13] The proportional hazard hypotheses were tested by Schoenfeld's residual method. The global proportional hazards assumption would not be met if we record significant associations (*p* < 0·05) for all correlation coefficients. Model discrimination was determined using a censor-adjusted c-statistic. Model calibration was demonstrated with a calibration plot generated using bootstrap resampling (*n* = 10,000), and the area under the receiver operating characteristic curve (AUC) was determined. Internal validation was performed using a penalised Cox model by adaptive elastic-net regularisation, which can outperform Lasso on data with highly correlated predictors. [14] Tenfold repeated cross-validation was used, which is a more robust internal validation method than bootstrapping (Supplementary Fig. 2). [15] The model utility was assessed using decision curve analysis [16]. A nomogram was developed, which incorporated the clinical predictive factors included in the final model. All statistical analyses were performed using R language, version 3.2.1 (R Foundation for Statistical Computing, Vienna, Austria).[17].

## Results

**Fig. 1:**
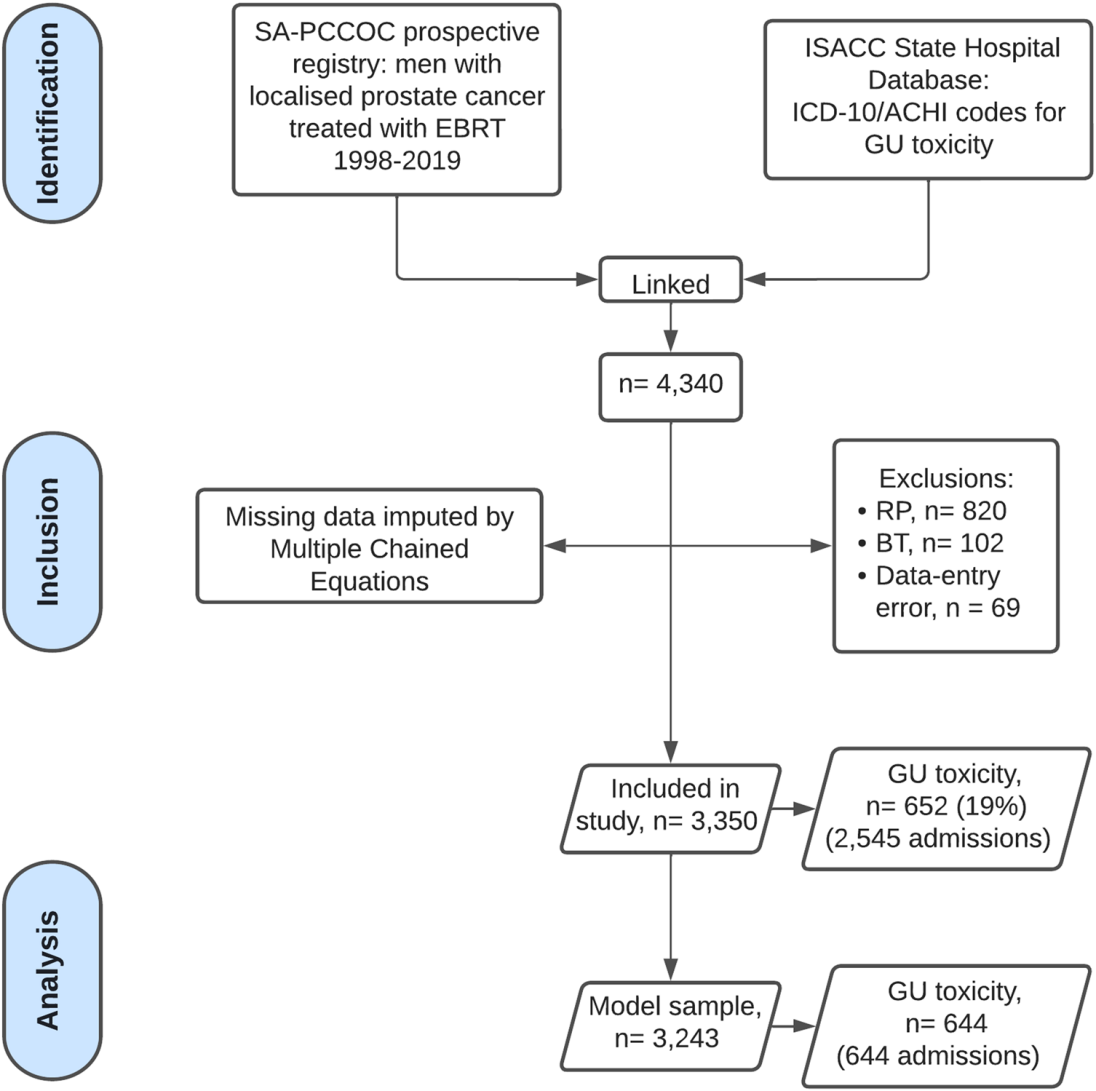
STROBE flow chart of patient selection

There were 3,243 patients with localised prostate cancer treated with curative intent radiotherapy included in the modelling dataset (Fig. 1). Table 1 outlines the patient baseline characteristics. Patients with BOO without TURP had the lowest 10 year EFS rates (20% [95% CI 15–27%], p < 0.001; Supplementary Table 3, Supplementary Fig. 3).

**Table 1:** Demographic and treatment baseline characteristics of included patient in model

		GUT Admission		*p*-value^2^
Characteristic	Overall, *N* = 3243^1^	No, *N* = 2599^1^	Yes, *N* = 644^1^	
Age				0.7
Median (IQR)	71 (66, 76)	71 (65, 76)	72 (66, 75)	
Range	43, 91	43, 91	47, 86	
Diabetes				** < 0.001**
No	2,611 / 3243 (81%)	2,148 / 2599 (83%)	463 / 644 (72%)	
Yes	632 / 3243 (19%)	451 / 2599 (17%)	181 / 644 (28%)	
Smoking				** < 0.001**
No	1,710 / 3243 (53%)	1,520 / 2599 (58%)	190 / 644 (30%)	
Yes	1,533 / 3243 (47%)	1,079 / 2599 (42%)	454 / 644 (70%)	
BOO and TURP				** < 0.001**
No	2,838 / 3243 (88%)	2,381 / 2599 (92%)	457 / 644 (71%)	
Yes	405 / 3243 (12%)	218 / 2,599 (8.4%)	187 / 644 (29%)	
BOO no TURP				** < 0.001**
No	3,008 / 3,243 (93%)	2,540 / 2,599 (98%)	468 / 644 (73%)	
Yes	235 / 3,243 (7.2%)	59 / 2,599 (2.3%)	176 / 644 (27%)	
No BOO no TURP				** < 0.001**
No	640 / 3,243 (20%)	277 / 2,599 (11%)	363 / 644 (56%)	
Yes	2,603 / 3,243 (80%)	2,322 / 2,599 (89%)	281 / 644 (44%)	
iNCCN risk category				**0.002**
High	1,477 / 2,915 (51%)	1,182 / 2,341 (50%)	295 / 574 (51%)	
Intermediate	1,054 / 2,915 (36%)	873 / 2,341 (37%)	181 / 574 (32%)	
Low	384 / 2,915 (13%)	286 / 2,341 (12%)	98 / 574 (17%)	
(Missing)	328	258	70	
Radiotherapy dose, Gy				** < 0.001**
1. < 74	1,414 / 3,243 (44%)	1,064 / 2,599 (41%)	350 / 644 (54%)	
2. ≥ 74	1,829 / 3,243 (56%)	1,535 / 2,599 (59%)	294 / 644 (46%)	
Radiotherapy date				** < 0.001**
< 2009	1,236 / 3,243 (38%)	823 / 2,599 (32%)	413 / 644 (64%)	
≥ 2009	2,007 / 3,243 (62%)	1,776 / 2,599 (68%)	231 / 644 (36%)	
Follow-up, years				** < 0.001**
Median (IQR)	1,945 (766, 3,291)	2,094 (836, 3,463)	1,336 (582, 2,567)	
Range	2, 7,553	3, 7,553	2, 6,716	

^1^n / N (%)

^2^Wilcoxon rank-sum test; Pearson's Chi-squared test

^*^Missing data imputed by multiple chained equations: Radiotherapy dose, Gy (n = 311 [no event: 236; event: 75]), fractions, (n = 311 [no event: 234; event: 77])

*GU* genitourinary, *TURP* transurethral resection of prostate, *PSA* prostate-specific antigen, *NCCN* National Comprehensive Cancer Network, *BOO* bladder outlet obstruction, *Gy* grey

**Fig. 2:**
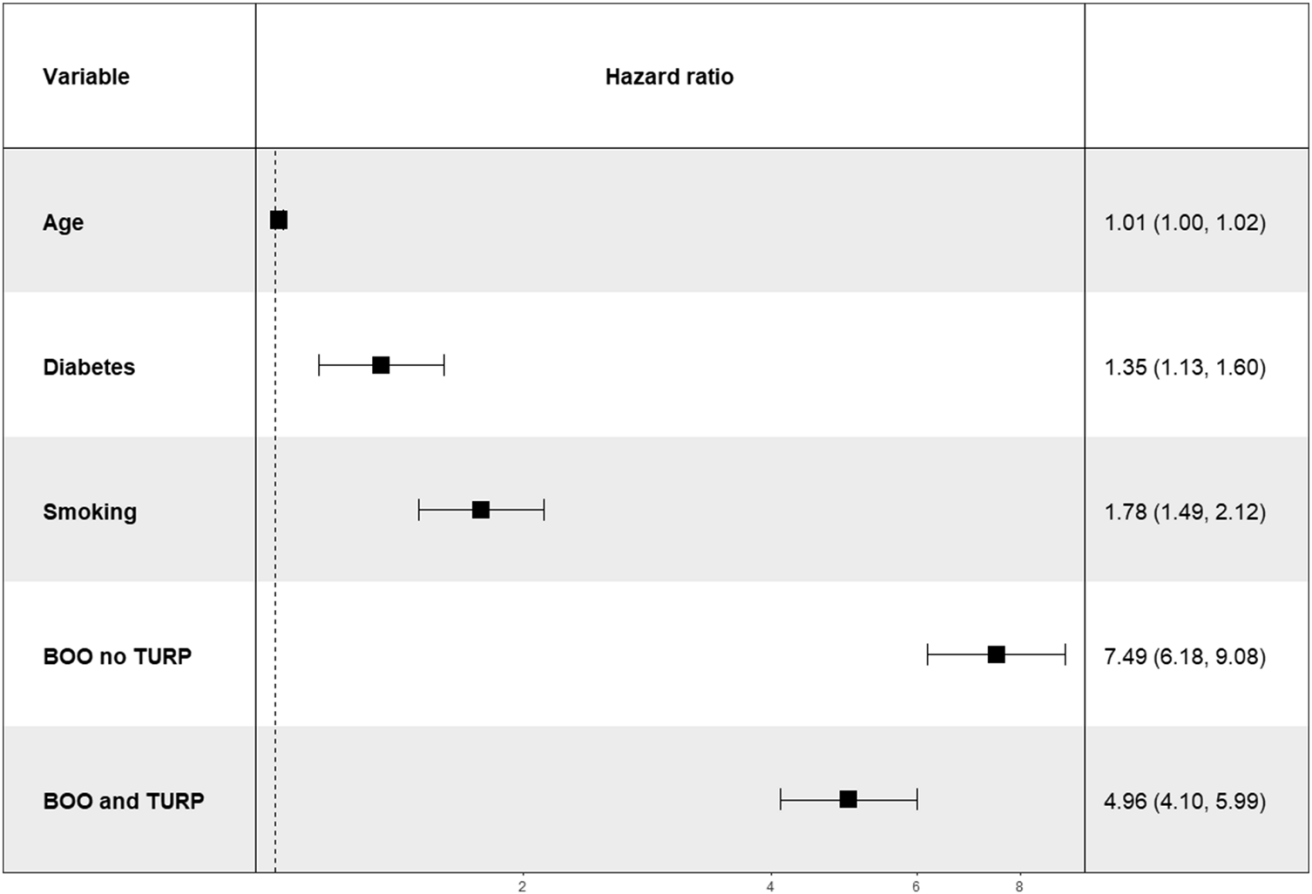
Forest plot of significant covariates in multivariate Cox regression model. * BOO no TURP and BOO and TURP were both calculated against no BOO no TURP as a reference in the multivariable analysis

After adjusting for age, multivariable analysis revealed diabetes (HR 1.35, 95% CI 1.13–1.60, *p* < 0.001), smoking (HR 1.78, 95% CI 1.40–2.12, *p* < 0.001), and BOO without TURP (HR 7.49, 95% CI 6.18–9.08 *p* < 0.001) followed by BOO with TURP ((HR 4.96, 95% CI 4.10–5.99 *p* < 0.001), to be strong independent predictors of hospitalisation for treatment-related genitourinary toxicity (Fig. 2). Baseline stress urinary incontinence was a strong independent predictor in multivariable analysis (HR 3.95, 95% CI 3.28–4.75, *p* < 0.001) but failed to meet the Schoenfeld proportional hazards test (*p* < 0.0001), with suspected multicollinearity with BOO and TURP and was therefore removed from the final model.

The final model met the proportional hazards with a global Schoenfeld test *p* = 0.1762.

**Fig. 3:**
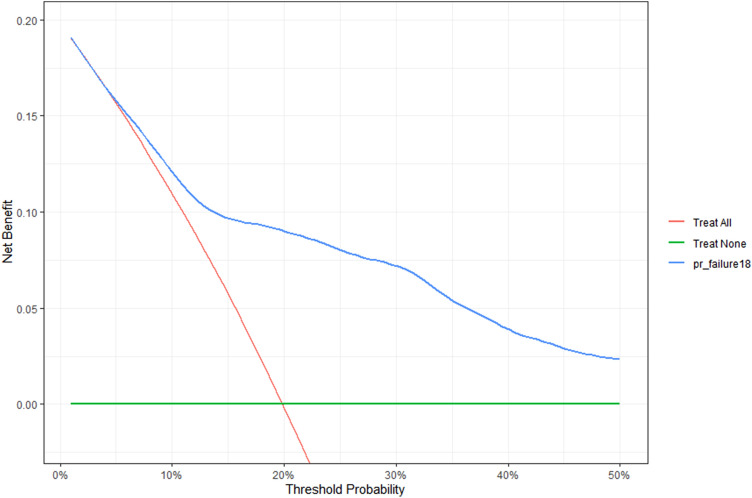
Decision curve analysis for radiotherapy-related genitourinary toxicity-related hospital admission using a Cox multivariable model

**Fig. 4:**
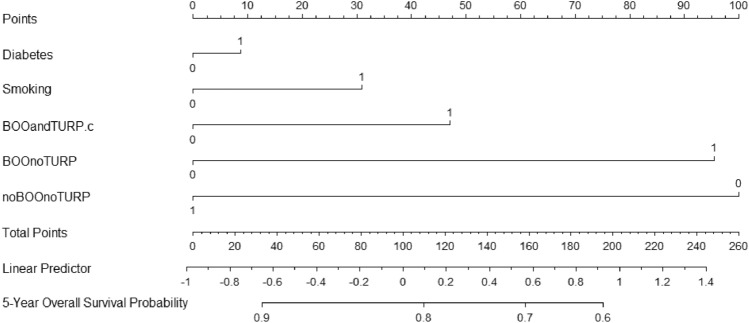
Nomogram for the prediction of genitourinary toxicity-related hospital admission post-EBRT for localised prostate cancer

The predictive model performed well with a censor-adjusted c-statistic of 0.80. The model was reasonably discriminate at 1 (AUC 0.765) and five years (AUC 0.75), (Supplementary Fig. 3) and was internally validated (Supplementary Fig. 4). The decision curve analysis determined the model's utility, with a consistently greater net benefit to patients with prostate cancer at risk of radiation-induced genitourinary from threshold probability > 5% (Fig. 3). A nomogram was developed to predict 5-year overall genitourinary toxicity event-free survival (Fig. 4).

## Discussion

This is the first study to develop a data-driven predictive model for treatment-related genitourinary toxicity requiring hospitalisation using pre-treatment clinical characteristics amongst patients with localised prostate cancer treated by curative intent EBRT. This involved the analysis of a prospectively state population-level cohort of patients (*n* = 3243) with an adequate median length of follow-up (5 years), which provided valuable information regarding predictive factors for the development of treatment-related genitourinary toxicity. With the selection of hospitalisation for treatment-related toxicity as an endpoint, the model can be compared with grade 3 RTOG/ CTCAE toxicity reported in the literature. The model performed strongly in calibration at one (AUC 0.765) and five years (AUC 0.75) (Supplementary Fig. 3). In addition, the model was discriminate (concordance index = 0.67, censor-adjusted c-statistic = 0.80) and is consistent with the most robust models in the literature, including the study by Yahya 2015 (concordance index 0.548–0.780). [5].

This was also the first predictive study for genitourinary toxicity requiring hospitalisation to include decision curve analysis (Fig. 3) and a nomogram (Fig. 4). The decision curve analysis consistently demonstrated net benefit in using the model compared to the treat-all approach above 5% threshold probability. The reliable prediction of radiotherapy-related toxicity amongst patients with prostate cancer has been valued by numerous other authors because it could guide the allocation of patients into treatment groups based on their probability of severe toxicity and improve the therapeutic ratio [18–20]. Patients at high risk of radiotherapy-related toxicity could be counselled about treatment alternatives, modifications (e.g. advanced planning corrections or dose-reduction) or deferrals. Although not statistically framed to address the question, our analysis also revealed that TURP prior to radiotherapy in patients with BOO might reduce the hazards of GU toxicity requiring admission [HR 7.49 (95% CI 6.18–9.08) vs HR 4.96 (95% CI 4.10–5.99)].

Few other models utilise the clinical characteristics of patients with prostate cancer treated with radiotherapy to predict post-treatment toxicity. Most other models were developed in small cohorts with few toxicity-related events (*n* < 500) [19, 21, 22]. In addition, given the plethora of complex biophysical manifestations of genitourinary toxicity that can develop, other investigators focus on different toxicity outcomes: early [23] vs late [5, 6, 19] toxicity, mild or severe toxicity (based upon variable grading systems [24], RTOG/EORTC [25, 26], CTCAE [21, 26], LENT-SOMA [5, 6, 9]) or specific symptoms [6, 26, 27] including haematuria [19, 21, 26], nocturia [19], IPSS [20, 23] and erectile dysfunction [28]. These perhaps have less observable impacts on the health system than hospital admissions, the outcome we have used.

Furthermore, very few predictive studies meet the TRIPOD criteria for reporting. There was inconsistent reporting of concordance index, with some reporting concordance probability estimates [9] and others AUC [5, 19, 26], creating difficulties comparing model calibration across studies. Other models were also less discriminative [5, 19, 26]. The calibration plot included in the current study appears as well calibrated as others presented in the literature. [6, 20] No other studies reported a c-statistic. Only one predictive model was externally validated [28]. Whilst other models often failed to report optimism [21, 26], we used a penalised Cox model by adaptive elastic-net regularisation.

Our study has several limitations. Firstly, we did not analyse radiotherapy delivery technique (i.e. 3D-CRT IMRT, VMAT, IGRT), field size or dose-volume effect, as these data were unavailable in the current study and have already been described [8, 9]. However, the majority of included patients were treated with EBRT ≥ 2009 (62%), indicating mostly contemporary treatment techniques. Furthermore, the included clinical predictive factors remained significant in multivariable analysis adjusted for year of treatment, as demonstrated in our recently published article [29]. In addition, we do not have information regarding baseline IPSS, prostate volume or 5-ARI or alpha-blocker medication use before EBRT. Similarly, we do not have information about whether patients received androgen deprivation therapy; however, we acknowledge that the impact of hormone therapy cannot accurately be determined given the bias to treat more unfavourable patients with hormone therapy. Finally, whilst the lack of external validation limits the generalisability of the study results, this is mitigated by using a prospectively captured state-population level dataset.

## Conclusion

This study demonstrates the feasibility of predicting radiotherapy-related genitourinary toxicity requiring hospitalisation utilising pre-treatment clinical characteristics for men with localised prostate cancer. Clinicians in the pre-operative counselling setting could use our nomogram to inform patient selection and treatment-related toxicity. TURP before EBRT partially reduces the risk of genitourinary toxicity for men with prostate cancer and bladder outlet obstruction, and this relationship requires further prospective scrutiny.

## Supplementary Information

Below is the link to the electronic supplementary material.

Supplementary file1 (DOCX 18 KB)

Supplementary file2 (PNG 24 KB)

Supplementary file3 (PNG 101 KB)

Supplementary file4 (PNG 15 KB)

Supplementary file5 (PNG 16 KB)

## Data Availability

The data that support the findings of this study are available from the corresponding author upon reasonable request.
