# Supplementary material for: Predicting post-radiation genitourinary hospital admissions in patients with localised prostate cancer
Source: World J Urol. 2022 Nov 10;40(12):2911–8. doi: 10.1007/s00345-022-04212-y (PMC9712379; doi:10.1007/s00345-022-04212-y)
Supplement: Supplementary file 1 — Supplementary file1 (DOCX 18 KB) [file 345_2022_4212_MOESM1_ESM.docx]

## Supplementary Table 1. Genitourinary Toxicity Admission Codes.

| **DESCRIPTION** | **ACHI Code** |
| --- | --- |
| Haematuria | R31 |
| Recurrent and persistent haematuria | N02 |
| Recurrent & persistent haematuria other | N028 |
| Recurrent & persistent haematuria unsp | N029 |
| Irradiation cystitis | N304 |
| Congestion and haemorrhage of prostate | N421 |
| Urethral stricture | N35 |
| Other urethral stricture | N358 |
| Urethral stricture unspecified | N359 |
| Other disorders of urethra | N36 |
| Urethral disorder unspecified | N369 |
| Oth atresia stenos urethra bladder neck | Q643 |
| Oth atrs stenos urethra & bladder neck | Q6439 |
| Postprocedural urethral stricture | N991 |
| Injury of urethra | S373 |
| Bladder-neck obstruction | N320 |
| Retention of urine | R33 |
| Mech comp urinary (indwelling) catheter | T830 |
| Stress incontinence | N393 |
| Other specified urinary incontinence | N394 |
| Unspecified urinary incontinence | R32 |

Note: Bladder outlet obstruction (BOO), was divided into four categories (i.e., + BOO + TURP, + BOO – TURP, - BOO + TURP, -BOO – TURP). Patients who underwent a TURP were then combined (BOOandTURP.c) regardless of whether administrative codes for BOO were present or not, given the clinical assumption that BOO would have been present and the missing flag would have been related to a coding error.

## Supplementary Table 2. Kaplan-Meier Event-Free Survival Estimates for Genitourinary Toxicity Admission Following Primary EBRT

| Characteristic | 5 Year | 10 Year | p-value^1^ |
| --- | --- | --- | --- |
| Overall | 85% (84%, 87%) | 72% (69%, 74%) |  |
| Age, years |  |  | **0.041** |
| 1.<75 | 86% (84%, 87%) | 73% (70%, 75%) |  |
| 2.≥75 | 83% (81%, 86%) | 69% (65%, 73%) |  |
| Charlson Score |  |  | **0.010** |
| 1. <2 | 82% (80%, 84%) | 66% (62%, 69%) |  |
| 2. >=2 | 76% (71%, 81%) | 57% (50%, 66%) |  |
| Diabetes Mellitus Type 2 |  |  | **<0.001** |
| No | 87% (85%, 88%) | 75% (72%, 77%) |  |
| Yes | 79% (76%, 83%) | 60% (55%, 66%) |  |
| Smoking History |  |  | **<0.001** |
| No | 91% (90%, 93%) | 83% (81%, 86%) |  |
| Yes | 79% (77%, 81%) | 60% (57%, 63%) |  |
| Stress Urinary Incontinence |  |  | **<0.001** |
| No | 89% (87%, 90%) | 78% (76%, 80%) |  |
| Yes | 45% (38%, 52%) | 10% (6.5%, 15%) |  |
| BOO no TURP |  |  | **<0.001** |
| No | 88% (86%, 89%) | 77% (75%, 79%) |  |
| Yes | 56% (50%, 63%) | 20% (15%, 27%) |  |
| BOO and TURP |  |  | **<0.001** |
| No | 89% (87%, 90%) | 77% (75%, 79%) |  |
| Yes | 60% (55%, 66%) | 36% (29%, 43%) |  |
| No BOO no TURP |  |  | **<0.001** |
| No | 59% (55%, 63%) | 29% (24%, 34%) |  |
| Yes | 92% (91%, 93%) | 84% (82%, 86%) |  |
| Radiotherapy dose, Gy |  |  | 0.7 |
| 1.<74 | 85% (83%, 87%) | 71% (68%, 74%) |  |
| 2.≥74 | 85% (83%, 87%) | 72% (69%, 75%) |  |
| NCCN Risk |  |  | **<0.001** |
| High | 84% (82%, 86%) | 70% (66%, 73%) |  |
| Intermediate | 87% (85%, 89%) | 77% (74%, 81%) |  |
| Low | 83% (79%, 87%) | 64% (58%, 71%) |  |
| EBRT completion date |  |  | **<0.001** |
| < 2009 | 82% (80%, 85%) | 68% (65%, 70%) |  |
| ≥ 2009 | 88% (86%, 89%) | 79% (76%, 82%) |  |
