# Supplementary figures and images for: Predicting post-radiation genitourinary hospital admissions in patients with localised prostate cancer
Source: World J Urol. 2022 Nov 10;40(12):2911–8. doi: 10.1007/s00345-022-04212-y (PMC9712379; doi:10.1007/s00345-022-04212-y)

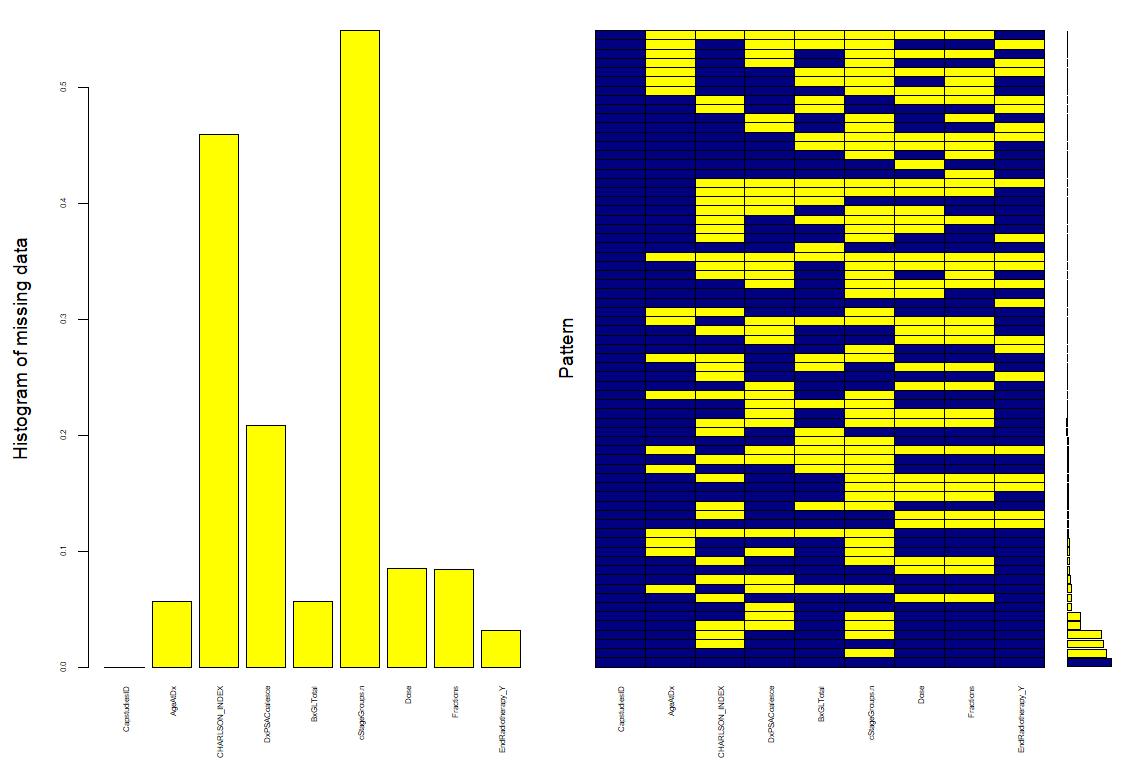

Supplement: Supplementary file 2 — Supplementary file2 (PNG 24 KB) [file 345_2022_4212_MOESM2_ESM.png]

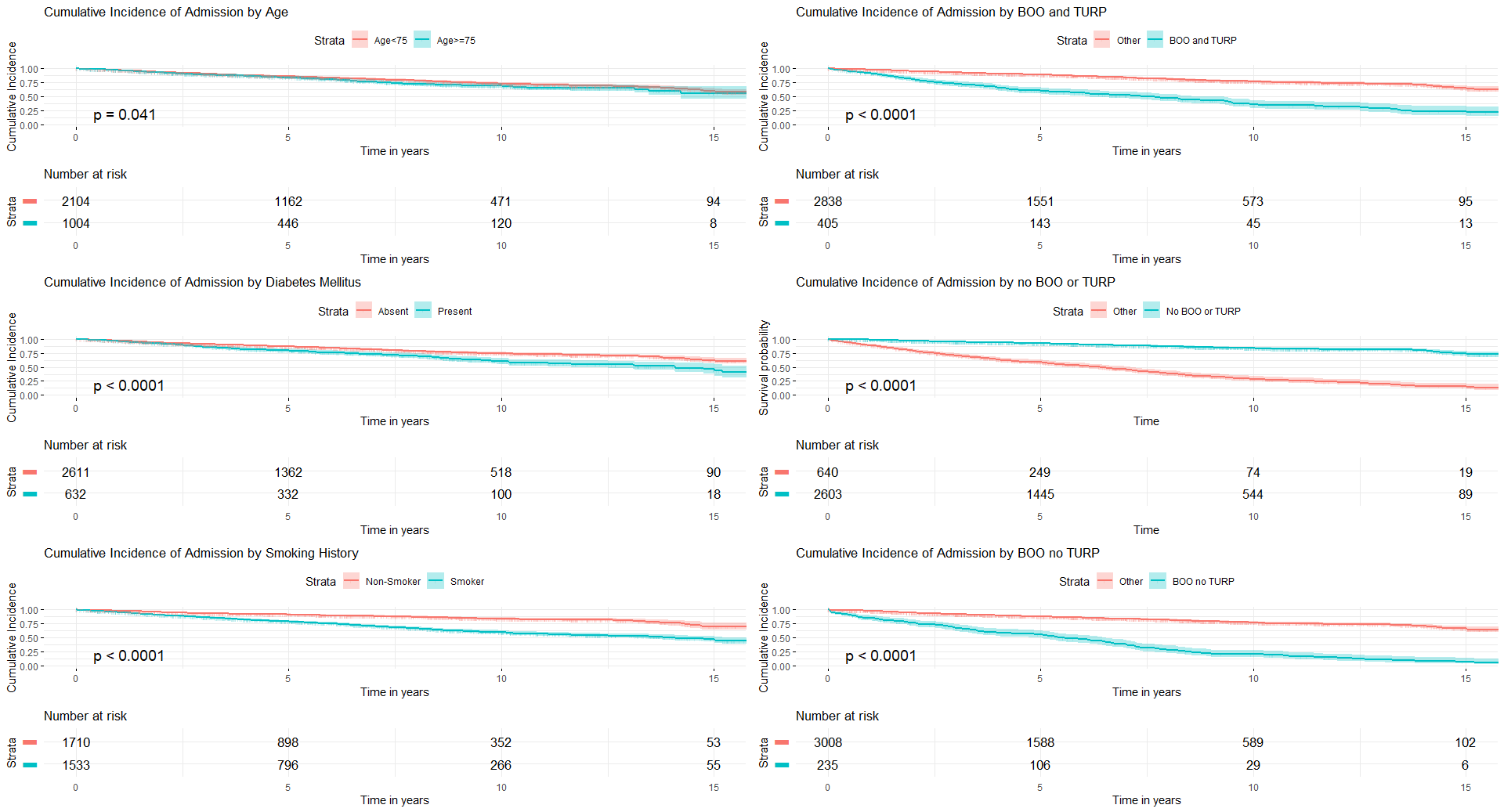

Supplement: Supplementary file 3 — Supplementary file3 (PNG 101 KB) [file 345_2022_4212_MOESM3_ESM.png]

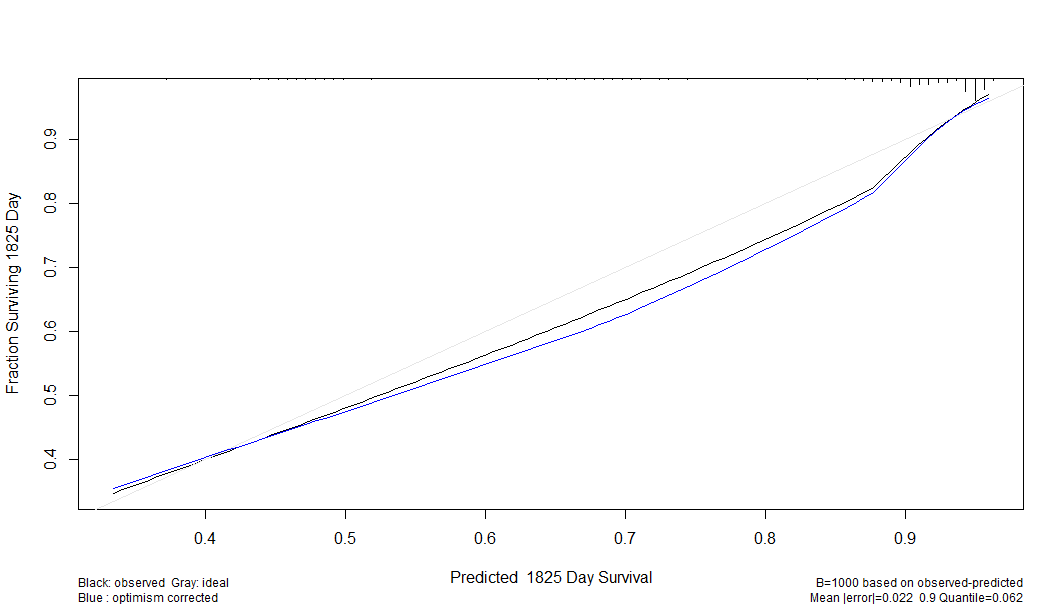

Supplement: Supplementary file 4 — Supplementary file4 (PNG 15 KB) [file 345_2022_4212_MOESM4_ESM.png]

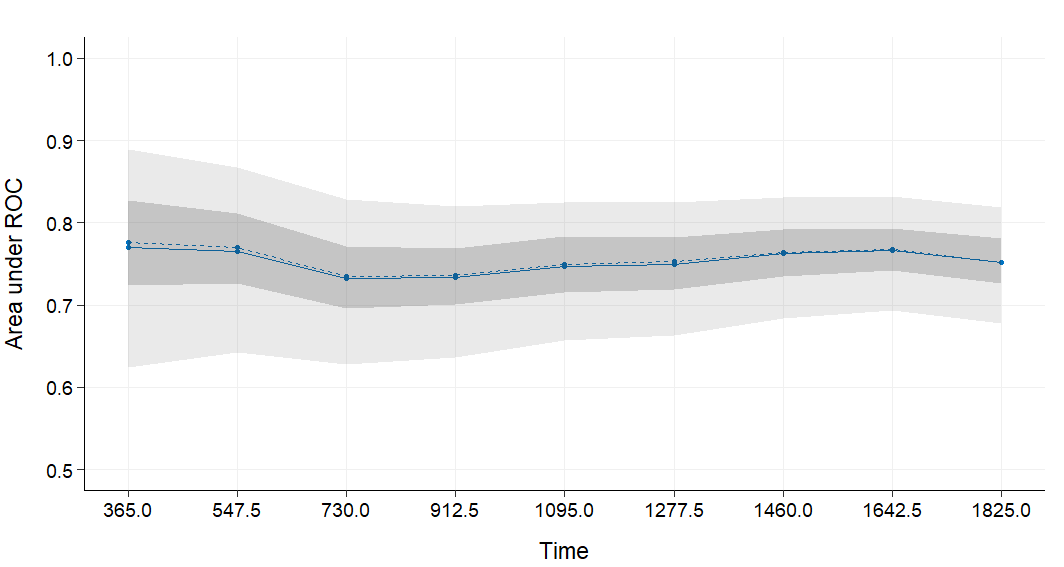

Supplement: Supplementary file 5 — Supplementary file5 (PNG 16 KB) [file 345_2022_4212_MOESM5_ESM.png]
